# Systematic comprehensive geriatric assessment in elderly patients on chronic dialysis: a cross-sectional comparative and feasibility study

**DOI:** 10.1186/1471-2369-13-30

**Published:** 2012-05-30

**Authors:** Juliette L Parlevliet, Bianca M Buurman, Marja M Hodac Pannekeet, Els M Boeschoten, Lucia ten Brinke, Marije E Hamaker, Barbara C van Munster, Sophia E de Rooij

**Affiliations:** 1 Academic Medical Center, University of Amsterdam, Department of Internal Medicine, Section of Geriatric Medicine, Room F4-108, Meibergdreef 9, 1105, AZ, Amsterdam, the Netherlands; 2 Zaans Medical Centre, Department of Internal Medicine, Koningin Julianaplein 58, 1502, DV, Zaandam, the Netherlands; 3 Hans Mak Institute, Wilhelminalaan 29-B, 1411, EL, Naarden, the Netherlands

## Abstract

**Background:**

Elderly dialysis patients are prone to disabilities and functional decline. This aggravates their last period of life. It would be valuable to be able to preserve daily function and quality of life. Identification of domains requiring additional attention is not common practice in standard care. Therefore, we performed a systematic Comprehensive Geriatric Assessment (CGA) to assess physical and psychosocial function and tested its feasibility in daily practice. The CGA is used more frequently in the assessment of elderly cancer patients, and we therefore compared the outcomes to this group.

**Methods:**

A cross-sectional, multicenter study, between June 1^st^ and September 31^st^, 2009, in four Dutch outpatient dialysis units. Fifty patients aged 65 years or above who received dialysis because of end-stage renal disease (ESRD) were randomly included. We assessed the CGA during a systematic interview with patients and their caregivers. The cancer patients had had a similar CGA in an earlier study. We compared prevalences between groups.

**Results:**

In the dialysis population (68.0% 75 years or above, 76.6% on haemodialysis) caregivers often observed behavioral changes, such as deviant eating habits (34.0%) and irritability (27.7%). In 84.4%, caregivers felt overburdened by the situation of their family member. Somatic and psychosocial conditions were frequently found (polypharmacy (94.6%), depression (24.5%)) and prevalence of most geriatric conditions was comparable to those in elderly cancer patients.

**Conclusions:**

Geriatric conditions were highly prevalent among elderly dialysis patients and prevalences were comparable in both populations. The CGA proved feasible for recognition of these conditions and of overburdened caregivers. This could prevent further functional decline and preserve quality of life.

## Background

In nephrology, a growing number of elderly patients receive renal replacement therapy because of end-stage renal disease (ESRD) [1]. Patients aged 75 years or more represent the fastest growing segment of the population starting dialysis [2-4]. This specific patient population is characterized by multi-morbidity, defined as the presence of two or more concomitant diseases, disabilities and geriatric conditions such as polypharmacy, sensory deficits, incontinence, low energetic falls, cognitive impairment and decreased social participation [3].

Functional decline, often defined as a deterioration in the activities of daily living (ADL), is a result of reduced physiological reserves [5]. Its presence in the elderly is often preceded or accompanied by geriatric conditions. The consequences of functional decline are decreased independence, lower quality of life, higher risk of institutionalization and death [6-9]. In ESRD, functional decline is observed in all patients, but elderly patients are at even higher risk than their younger counterparts [2,10-12]. In addition, the initiation of dialysis is associated with a substantial decline in functional status and dialysis patients are also more prone to develop cognitive impairment [2,13,14]. Consequently, for patients who are at increased risk of functional or cognitive decline, it is of the utmost importance that potential problems are recognized early to allow health care professionals to slow or prevent this decline [15]. From the patient’s perspective, information regarding the presence of geriatric conditions and their impact on daily function could assist in the decision-making process when considering the most appropriate form of renal replacement therapy or when accepting non-dialysis therapy [16].

The Comprehensive Geriatric Assessment (CGA) is widely used in geriatric care, but outside this specialization, a CGA is not often applied. In oncology, the employment of the CGA is gaining interest, primarily in research settings. A CGA could be useful to identify individual older adults with ESRD who are on the trajectory of (progressive) functional or cognitive decline or for benchmarking purposes.

The aim of this cross-sectional study was to perform a systematic CGA to investigate somatic, psychological, functional and social function in a group of older dialysis patients. Secondly, we aimed to place our findings in a broader perspective by comparing our group to a population of elderly cancer patients who likewise suffered from an end-stage chronic progressive disease. Finally, we asked the multidisciplinary team for their opinion on the feasibility of the systematic CGA and the relevance of its outcome.

## Methods

### Design, setting and participants

This cross-sectional study was conducted between June 1^st^ and September 31^st^, 2009, in four Dutch hospitals with dialysis facilities. Zaans Medical Centre, Zaandam; Westfries Gasthuis, Hoorn; and Tergooi Hospitals, Hilversum are teaching hospitals; the Academic Medical Center, Amsterdam is a tertiary university teaching hospital. All patients with ESRD aged 65 years or above, either receiving peritoneal dialysis or haemodialysis were eligible for participation. Patients were excluded if they had insufficient knowledge of the Dutch language. Nephrologists of the participating dialysis centres identified eligible patients, informed them personally and by patient information letter about the study and asked them for permission to be contacted by a research nurse from the Hans Mak Institute, an independent institute for quality management in the field of kidney diseases. If patients agreed, they were asked for written informed consent for participation in the study by this research nurse. The medical ethics committee of Zaans Medical Centre approved the study.

Data from the ESRD patients (n = 50) were compared to the data from a Dutch population of acutely admitted (non-selected and consecutive) cancer patients aged 65 years and older (n = 292), in which the same systematic CGA was performed. The methods and results of that study were published elsewhere [17].

### Data collection

Patients were visited at home by the research nurse between two dialysis sessions. Prior to the visit, she sent the patients two questionnaires by mail: one for the patient and one for the primary caregiver. During the home visit, she completed the postal questionnaires in a face-to-face interview and conducted assessments of cognition, decubitus and delirium. Data on the general demographics, dialysis and co morbidities of each patient were retrieved from the hospitals’ medical charts.

### Systematic CGA

The systematic Comprehensive Geriatric Assessment consisted of various validated tests and questionnaires on four domains of patient function: the somatic, psychological, functional and social domains. Table 1 summarizes the CGAs instruments used, including their cut-off values.

**Table 1:** Content of the systematic comprehensive geriatric assessment

**Geriatric conditions**	**Measurement tool**	**Source**	**Range of scores (cut-off) used**
*Somatic geriatric conditions*			
Polypharmacy	Number of different medications	medical chart	Ordinal, (≥ 5)
Malnutrition	Short Nutritional Assessment Questionnaire (SNAQ) [18]	patient	0 – 7, (≥ 2)
Obesity	Body Mass Index (BMI)	medical chart	Continuous, (>30)
Pain	Visual Analogue Scale (VAS) [19]	patient	0 – 10
Decubitus	Prevention and Pressure Ulcer Risk Score Evaluation (prePURSE) [20]	patient and nurse	0-46, (≥20)
Constipation	Constipation	patient	yes or no
Incontinence	Presence of incontinence	patient	yes or no
Falls	Two or more falls in the past three months	patient	yes or no
Co morbidity	Charlson co morbidity index [21]	medical chart	0–31
*Functional geriatric conditions*			
ADL functioning	Katz ADL index score [22]	patient and caregiver	0–6, (≥ 1)
IADL functioning	Modified Katz index [23]	patient and caregiver	0–9, (≥ 1)
Neurosensory deficits	Impairment of hearing and/or vision, regardless of use of glasses or hearing aid	patient	yes or no
Mobility	Requiring help or the use of a walking aid for mobility	patient	yes or no
Self-perceived Health status	EuroQol (EQ-6D) [24]	patient	6 items, (decreased if scored “severe” ≥ 1)
Quality of life	Visual Analogue Scale in EuroQol-6D [25]	patient	0–100
*Psychological geriatric conditions*			
Global cognitive state	Mini Mental State Examination (MMSE) [26]	patient	≤24/30
Cognitive impairment	Informant Questionnaire on Cognitive Decline in the Elderly-short form (IQCODE-SF) [27,28]	caregiver	16 items, score1 – 5, max score 80 (impairment if ≥ 63, or 3.9 (63/16))
Behavioural disturbances	Neuropsychiatric Inventory Questionnaire (NPI-q) [29]	caregiver	yes or no
Depressive symptoms	Geriatric Depression Scale-15 (GDS-15) [30]	patient	0 – 15, ≥6
Delirium	Confusion Assessment Method (CAM) [31]	nurse	0 – 4, item 1 and 2 plus 3 and/or 4
*Social geriatric conditions*			
Caregiver burden	Experienced Burden of Informal Care (EDIZ) [32]	caregiver	0 – 9, ≥ 4
Loneliness	De Jong-Gierveldschaal [33]	patient	≥3/11 indicates loneliness

### Feasibility of the CGA in daily practice

Feasibility of the CGA was assessed in an interview with the multidisciplinary team and in feedback panels. In each dialysis centre, a patient, a medical psychologist, a social worker, a nurse, and a nephrologist were interviewed by the research nurse about the relevance of the questionnaires’ content and the team’s need for screening instruments to assess elderly dialysis patients’ vulnerability. This interview mainly addressed acceptability and feasibility of the CGA to the team. Logistics and acceptability of the CGA for the dialysis patients were discussed in two feedback panels of elderly patients. The first feedback panel consisted of dialysis patients who also took part in the interview with the multidisciplinary team. The second feedback panel consisted of elderly who advise the research team of Geriatrics in the AMC on research questions.

### Statistical analysis

Statistical analyses were performed with SPSS software, version 16.0 (Statistical Package for the Social Sciences Inc., Chicago, IL). Standard descriptive statistics were used. Furthermore, dialysis patients were compared to elderly cancer patients for differences in co morbidities, polypharmacy and the outcomes of the systematic CGA. We tested for differences in the characteristics of the two populations using Student’s t-tests and Chi-squared tests.

## Results

### Characteristics of ESRD patients

Fifty dialysis patients and their primary caregivers were interviewed. Baseline characteristics are reported in Table 2. Female patients constituted 26.0% of the population, and 68.0% of all patients were older than 75 years. Haemodialysis was applied in 77.0%, and 23.0% received peritoneal dialysis. The most prevalent geriatric condition was polypharmacy (94.6%). Other frequently observed conditions were hearing impairment (36.8%), malnourishment (32.7%), social or emotional loneliness (30.6% combined) and depressive symptoms (24.5%). In this population, 24.0% of patients reported pain. (Instrumental) activities of daily life are presented in Table 3. The majority of difficulties were related to housekeeping, travelling and walking. The patients were relatively independent with regard to the basic activities of daily living, such as eating, toileting, bathing, dressing and walking.

**Table 2:** Baseline results, demographics of community dwelling elderly on chronic dialysis

**Variable**	**Patients n = 50**
**Age (%)**	
65–74 yr	32.0
75–84 yr	58.0
> 85 yr	10.0
Male (%)	76.0
Years of education (mean/SD)	11.0 (3.7)
**Highest level of education reached (%)**	
Primary school or lower vocational training	33.3
Vocational training	33.2
Higher vocational training	20.8
University	4.2
Other	8.5
**Dutch ethnicity (%)**	96.0
**Working situation (%)**	
Retirement	96.0
Employed	4.0
**Social status (%)**	
Single	40.0
Living with partner or child	60.0
**Living situation (%)**	
Independent	81.6
Other*	18.3
**Kind of dialysis (%)**	
Haemodialysis	76.6
Peritoneal dialysis	23.4
**Co morbidity**	
Charlson co morbidity index score** (mean/SD)	4.6 (2.3)

SD standard deviation.

*Living situation other: home for the elderly or transitional care.

**Range 0–31; a higher score indicates more and/or more severe co morbidities.

**Table 3:** (Instrumental) Activities of Daily Living according to the modified Katz ADL index

**Modified Katz ADL index question**	**% needs help**
1. Bathing	18.0
2. Dressing	10.0
3. Combing hair	4.0
4. Toileting	4.0
5. Continence	8.2
6. Transfer bed-chair	10.0
7. Walking	27.0
8. Eating	0.0
9. Telephoning	4.0
10. Travelling	33.0
11. Shopping groceries	18.0
12. Cooking	18.0
13. Household keeping	50.0
14. Taking medication	0.0
15. Managing finances	6.0

On the visual analogue scale in the EuroQol-6D [25], patients rated their own health-related quality of life to have a mean score of 61.8 (range 0–100, SD 18.5), and 9.8% of patients scored having severe problems on one or more item of the EuroQol-6D. Co morbidity was highly prevalent, with a mean Charlson co morbidity index of 4.6 points (SD 2.3).

The results of the Neuropsychiatric Index (NPI) are shown in Table 4. Caregivers reported a number of behavioural problems, of which changes in appetite or eating behaviour were most prevalent (34.0%). In addition, depression or dysphoria, apathy, and irritability or emotional lability were all reported in over a fourth of our population. In 84.4% of patients, caregivers experienced care as a very large burden.

**Table 4:** Neuropsychiatric Inventory

**NPI**	**% yes**
Delusions	0.0
Hallucinations	2.0
Agitation/aggression	8.5
Depression/dysphoria	25.5
Anxiety	8.5
Euphoria	19.1
Apathy	25.5
Dis-inhibition	6.4
Irritability/lability	27.7
Aberrant motor behaviour	0.0
Night time behaviour disturbances	19.1
Change in appetite/eating behaviour	34.0

### Prevalence of geriatric conditions in dialysis patients versus cancer patients

In Table 5, the geriatric conditions in dialysis patients were compared with those of 292 hospitalized cancer patients. Age was comparable between the two cohorts. The mean age in the dialysis patients was 77.1 years (SD 6.8 years) versus 75.7 years (SD 7.5 years) in the cancer patients. Compared to cancer patients, more dialysis patients were male (74.0% versus 51.7% (p = 0.01)). In both groups, the majority lived independently (81.6% versus 83.0%) and with partner or child (58.8% versus 60.1%), but cancer patients more often lived in a nursing home (p = 0.01).

**Table 5:** Comparison of basic demographics and geriatric conditions between elderly dialysis patients and elderly cancer patients

**Variable**	**Dialysis Patients (N = 50)**	**Cancer Patients (N = 292)**	**p-value**
Age, mean (years) (95% CI)	77.1 (75.2–79.0)	75.7 (74.9–76.6)	0.23
Sex, male %	74.0	51.7	0.01
***Somatic geriatric conditions***			
Polypharmacy, %	94.6	48.0	<0.001
Moderately-severely malnourished %	32.7 (16/49)	46.0 (104/226)	0.09
Pain, %	24.0 (12/50)	64.8 (83/128)	<0.001
Decubitus, %	2.1 (1/47)	1.4 (2/139)	0.76
Constipation, %	6.3 (3/48)	22.1 (34/154)	0.001
Incontinence for faeces or urine, %	6.4 (3/47)	25.2 (67/266)	0.01
Falls (2 or more falls in last 3 months), %	10.4 (5/48)	12.7 (33/259)	0.65
Charlson co morbidity score, mean (95% CI)	4.6 (0.1–9.1)	5.6 (0.7–10.5)	
***Functional geriatric conditions***			
KATZ total”, mean (95% CI)	2.0 (1.2–2.8)	3.3 (2.9–3.6)	0.01
≥ 1 limitations Katz, %	61.7	79.1	0.01
ADL impairment”, %,	25.0 (12/48)	38.1 (106/278)	<0.001
mean, (95% CI)	0.5, (0.2–0.8)	0.8, (0.6–1.0)	
IADL impairment”, %,	59.6 (28/47)	76.9 (196/255)	0.01
mean, (95% CI)	1.5, (0.9–2.1)	2.4, (2.2–2.7)	
Neurosensory deficit, %	44.4 (12/27)	26.0 (71/273)	0.04
Use of walking device, %	31.3 (15/48)	47.9 (134/280)	0.03
Decreased health related quality of life, %	9.8	12.0	NA,^
Euroqol VAS, mean, (95% CI)	61.8 (56.5–67.1)	NA	NA
***Psychological geriatric conditions***			
Cognitive impairment (MMSE ≤ 24), %	6.7 (3/45)	NA, *	NA
IQCODE score, mean, (95% CI)	3.1(2.9–3.3) (N = 34)	3.3 (3.2–3.4) (N = 205)	0.09
Cognitive impairment (IQCODE), %	5.9 (2/34)	15.7 (31/197)	0.13
Depressive symptoms, %	24.5	21.3	NA,^^
Delirium, %	0.0	27.4 (62/288)	<0.001
***Social geriatric conditions***			
Informal caregiver overburdened, %	84.4 (38/45)	43.8 (49/112)	<0.001
Number of geriatric problems, mean (95%CI)	5.9 (5.3–6.5)	5.3 (4.9–5.7)	0.47
No geriatric conditions found, %	2 (1/51)	8.2 (24/295)	0.03

95%CI 95% confidence interval.

“KATZ total score 0–15; ADL impairment: KATZ- questions 1, 2, 4, 5, 6, 8: score 0–6; IADL impairment: KATZ- questions 9–15: score 0–7; higher score means more impaired.

NA Not applicable.

^Quality of Life was assessed in ESRD patients by EuroQuol-6D and in cancer patients by EuroQuol-5D.

*MMSE unreliable due to many delirious patients.

^^Depression was assessed in ESRD patients by GDS-15 and in cancer patients by GDS-2.

Polypharmacy was more prevalent in dialysis patients, and pain was more prevalent in cancer patients (both p < 0.001). There was a significant difference in ADL impairment: 25.0% of dialysis patients had one or more ADL impairments, while this percentage was 38.1% in cancer patients (p < 0.001). Despite this, the burden of care was higher in dialysis patients: 84.4% of informal caregivers of dialysis patients reported being overburdened compared to 43.8% for informal caregivers of cancer patients (p < 0.001).

In dialysis patients, neurosensory deficits were more prevalent compared to cancer patients. For the cohort of dialysis patients, a distinction was made between visual and hearing impairments, the latter of which was most prevalent (10.6% vs. 36.8%). Memory problems as recorded by the Mini Mental State Exam (MMSE) [26] were present in a high percentage in acutely admitted cancer patients (30.1%). Because of the concomitant high prevalence of delirium in these and because delirium influences MMSE scores, the MMSEs of these patients could not be compared to the MMSEs of ESRD patients. Global cognitive impairment as based on Informant Questionnaire Cognitive Decline – Short Form (IQCODE-SF) [27,28] score was present in 5.9% of the ESRD patients and in 15.7% of the cancer patients (p = 0.13).

On average, ESRD patients had 5.9 geriatric conditions (95% CI: 5.3–6.5), and 98.0% had one or more geriatric conditions. Cancer patients had 5.3 geriatric conditions on average (95% CI: 4.9–5.7), and 91.8% had one or more geriatric condition (p = 0.47).

### Feasibility of the CGA

In the dialysis centres where this study was conducted, the nurse’s regular, structured intake included an outline of existing problems. The questionnaires which were send to the patient and the care provider took one hour to complete, the interview by the research nurse at the patient’s home took another hour. This was considered time consuming both by professionals and by patients. Patients and care givers appreciated the time spent and the attention that was given to the impact of ESDR on daily functioning. Furthermore, patients and caregivers thought the CGA could help the professionals to deal with their problems more adequately. Although the multidisciplinary team thought the CGA was extensive and time-consuming, all questionnaires were considered useful, with the exception of the Prevention and Pressure Ulcer Risk Score Evaluation (prePURSE) [20] to assess the risk of pressure ulcers and the Confusion Assessment Method (CAM) [31] for diagnosing delirium. The structured information regarding caregiver burden and detailed information on behavioural problems and depressive symptoms was considered particularly valuable. Some issues were perceived important by professionals, but were not addressed in the questionnaires. This concerned patients living alone or whose caregiver was deceased in combination with the lack of cooperation with care facilities that was sometimes experienced and problems with patient transportation by taxi.

## Discussion

This systematic CGA of a cross-sectional cohort showed that geriatric conditions were highly prevalent in older ESRD patients. In addition to expected somatic problems, such as polypharmacy, malnourishment and hearing problems, many less anticipated problems in the psychosocial and functional domains were identified. Behavioural changes and disturbances were observed frequently and many caregivers felt overburdened by the care they provided to the primarily ADL-independent ESRD patient. Furthermore, depressive symptoms were highly prevalent, which have large impact on both patient and care giver. Geriatric conditions in both chronic diseases, ESRD and cancer, were comparable in terms of the number of geriatric problems, but they differed significantly in the rate of ADL-impairment, the burden of caregivers and pain score. The multidisciplinary nephrology team considered the CGA to be extensive and informative.

This study is a contribution to the growing number of studies addressing geriatric conditions in ESRD patients [13,15,34-36]. All these studies emphasize different aspects of geriatric conditions in ESRD patients. To our knowledge, this study is the first to use the instrument of a CGA to systematically address all relevant geriatric domains. In addition to their ESRD, our patients faced an average of six geriatric conditions. These problems were likely to influence health and quality of life. The awareness of these problems by health care providers can facilitate deceleration and prevention of further decline in these patients [12,34,37]. Furthermore, interventions that have limited impact on the expense and efficiency of care are available for several of the geriatric conditions identified [38]. An assessment like this systematic CGA and appropriate training to manage the identified geriatric conditions should be introduced simultaneously to improve patient outcomes. Although nephrology care units generally use a multidisciplinary approach for all patients, our study demonstrates that in the geriatric dialysis population, more attention is needed for the important and burdened role of caregivers. As only 25% of ESRD patients had one or more ADL-impairments, the burden of caregivers must primarily be due to other causes. Our hypothesis is that frequent dialysis treatments, changes in physical and mental capacity and the behavioural disturbances of their family member are important factors. The rate of depressive symptoms we found, 24.5%, is consistent with other studies, which state rates between 20 and 35% [13,39]. Behavioural changes such as depressive symptoms, apathy and irritability can all be manifestations of a depression and can weigh disproportionately heavily on caregivers. In addition, loneliness was highly prevalent in the dialysis patients. Therefore, one goal of the multidisciplinary team should be to support caregivers in their task and thus prevent the social isolation of both caregivers and patients. This goal’s importance is emphasized by the fact that social support and embedding are predictive factors for treatment success and mortality in ESRD patients [34,40,41].

This is the first study comparing ESRD patients to a group of elderly cancer patients. As in oncology, it may be useful to determine which factors influence the outcome and burden of treatment because treatment in ESRD is similarly intensive, expensive and has important side effects influencing health-related quality of life [42]. Earlier studies demonstrated that geriatric conditions were predictive of poorer health outcomes in both older ESRD and older cancer patients in outpatient settings [34,35,41,43-45]. In the present study, we have demonstrated that these geriatric conditions are equally prevalent in cohorts of ESRD and acutely hospitalized older cancer patients. We are convinced that the conclusion of Rao et al., who found that for older patients with cancer, geriatric care improved quality of life, likewise applies for elderly ESRD patients [46].

Some limitations of the study should be stated. First, the number of participants is small and may not be representative, which might make extrapolation to all older dialysis patients less convincing. Participants were asked by their nephrologists to participate. This may have caused some selection bias because physicians might be more reluctant to ask sicker patients or patients with major cognitive impairment to enrol. As a result, it is possible that our population reflects relatively healthy patients. This would imply that the outcome of a similar study in the total dialysis population might be even worse. On the other hand, even these ‘healthy’ patients experienced a large burden of unrevealed geriatric conditions, and they were comparable to acutely admitted cancer patients. For the aim of this study, which was to explore the feasibility of a new method, this possible selection bias is less relevant. In general, ESRD is associated with an increased risk of cognitive impairment, and the prevalence of both cognitive impairment and dementia is higher than in the general population [2,14]. Again, however, the selection process may have resulted in a lower than expected prevalence of these problems in our cohort.

Furthermore, when examining our cohort composition, it appeared that a relatively small number of women participated in the study; however, in comparing our rate to other studies, it is apparent that gender varies widely in ESRD study populations and that our study is no exception [13,38].

Our study cannot identify associations between geriatric conditions in elderly dialysis patients and their risk for poor outcomes in dialysis. This knowledge would be useful for planning care in advance and, when made available at earlier stages, could inform and assist patients and their caregivers in making decisions regarding treatment options in ESRD [3,15,38]. We have demonstrated that a large proportion of our patients required aid in their daily activities, and this requirement is likely to increase during ongoing treatment [13,47]. The burden of care experienced by caregivers was large, but our questionnaires were insufficient to support a more detailed understanding of the specific reasons behind this burden. Another limitation of our study is the comparison of outpatient dialysis patients with hospitalized cancer patients. It is likely that the cancer patients were more severely ill than the dialysis patients. In particular, the different scores on the item ‘pain’ and the Charlson co morbidity index score may be influenced by the difference in acute illnesses. However, despite this discrepancy, the spectrum of the conditions is informative regarding the geriatric conditions that the dialysis staff must anticipate. Finally, for this study, we applied the CGA at all patients who were eligible to participate. Although feasible for both patients, care givers and professionals, it was rather time consuming. This time was well spend for some patients, but less appropriate for others. For the patients in which most problems were found with the CGA, the CGA was probably most burdensome. On the other hand, this was not mentioned by them to the research nurse and the time spend on the CGA was well appreciated by the patient and their care givers. In future, we would like to enhance efficiency by finding methods to screen patients, to select dialysis patients for which a CGA is especially useful. This might also enhance support for the CGA in dialysis centres.

The strength of our study is that it demonstrates the feasibility and significance of a systematic CGA in dialysis patients, while at the same time revealing issues that are not yet covered in highly organized standard care. The multidisciplinary dialysis teams stated in their interview that questionnaires on social and psychological problems were especially informative. These could be added to the standard procedure in order to gather potential cues for improving care and quality of life. This study highlights the usefulness of general geriatric principles in offering multidimensional, holistic care to chronic patients on dialysis and achieving a balance between these principles and the more technical, highly efficient care offered in nephrology [38].

In the future, we would like to perform a prospective study on the efficiency and the effects of a systematic CGA on the outcome of dialysis treatment and on quality of life.

## Conclusion

Elderly dialysis patients are prone to disabilities and functional decline, which aggravate their last period of life. It would be valuable to be able to prevent further functional decline and to preserve quality of life. In this study, we tested a systematic comprehensive geriatric assessment for this purpose. Our systematic comprehensive geriatric assessment proves feasible to specify potentially modifiable problems and geriatric conditions that can decrease quality of life and that are easily missed if not specifically anticipated. Also, we conclude that elderly dialysis patients have a high number of geriatric conditions and that they are comparable to acutely hospitalized elderly cancer patients with regard to geriatric conditions, as an equally vulnerable population.

## Competing interests

The authors declare that they have no competing interests.

## Authors’ contributions

JP, BB, MH, EB, LB and contributed to conception and design, or acquisition, analysis or interpretation of data, or both. JP drafted the article. JP, BB, MH, EB, MH, BM, SR provided intellectual content to the work described. All participated in revising the article and approved of the version to be published. All authors read and approved the final manuscript.

## Pre-publication history

The pre-publication history for this paper can be accessed here:

http://www.biomedcentral.com/1471-2369/13/30/prepub

## References

[B1] Stack AG Messana JM Renal replacement therapy in the elderly: medical, ethical, and psychosocial considerations Adv Ren Replace Ther 2000 7 52 62 10672917 10.1016/s1073-4449(00)70006-9

[B2] Cavalli A Del Vecchio L Locatelli F Geriatric nephrology J Nephrol 2010 23 11 15 20872365

[B3] Kurella M Covinsky KE Collins AJ Chertow GM Octogenarians and nonagenarians starting dialysis in the United States Ann Intern Med 2007 146 177 183 17283348 10.7326/0003-4819-146-3-200702060-00006

[B4] Ronsberg F Isles C Simpson K Prescott G Renal replacement therapy in the over-80s Age Ageing 2005 34 148 152 10.1093/ageing/afi024 15713858

[B5] Buurman BM van Munster BC Korevaar JC de Haan RJ de Rooij SE Variability in measuring (instrumental) activities of daily living functioning and functional decline in hospitalized older medical patients: a systematic review J Clin Epidemiol 2011 64 619 627 10.1016/j.jclinepi.2010.07.005 21074969

[B6] Covinsky KE Palmer RM Counsell SR Pine ZM Walter LC Chren MM Functional status before hospitalization in acutely ill older adults: validity and clinical importance of retrospective reports J Am Geriatr Soc 2000 48 164 169 10682945 10.1111/j.1532-5415.2000.tb03907.x

[B7] Inouye SK Zhang Y Han L Leo-Summers L Jones R Marcantonio E Recoverable cognitive dysfunction at hospital admission in older persons during acute illness J Gen Intern Med 2006 21 1276 1281 10.1111/j.1525-1497.2006.00613.x 16965558 PMC1924736

[B8] Joray S Wietlisbach V Bula CJ Cognitive impairment in elderly medical inpatients: detection and associated six-month outcomes Am J Geriatr Psychiatry 2004 12 639 647 15545332 10.1176/appi.ajgp.12.6.639

[B9] Norman K Pichard C Lochs H Pirlich M Prognostic impact of disease-related malnutrition Clin Nutr 2008 27 5 15 10.1016/j.clnu.2007.10.007 18061312

[B10] Kurella TM Incidence, management, and outcomes of end-stage renal disease in the elderly Curr Opin Nephrol Hypertens 2009 18 252 257 10.1097/MNH.0b013e328326f3ac 19374012 PMC2738843

[B11] Schell JO Germain MJ Finkelstein FO Tulsky JA Cohen LM An integrative approach to advanced kidney disease in the elderly Adv Chronic Kidney Dis 2010 17 368 377 10.1053/j.ackd.2010.03.004 20610364 PMC4466119

[B12] Li M Tomlinson G Naglie G Cook WL Jassal SV Geriatric comorbidities, such as falls, confer an independent mortality risk to elderly dialysis patients Nephrol Dial Transplant 2008 23 1396 1400 18057068 10.1093/ndt/gfm778

[B13] Kurella TM Covinsky KE Chertow GM Yaffe K Landefeld CS McCulloch CE Functional status of elderly adults before and after initiation of dialysis N Engl J Med 2009 361 1539 1547 10.1056/NEJMoa0904655 19828531 PMC2789552

[B14] Kurella M Chertow GM Fried LF Cummings SR Harris T Simonsick E Chronic kidney disease and cognitive impairment in the elderly: the health, aging, and body composition study J Am Soc Nephrol 2005 16 2127 2133 10.1681/ASN.2005010005 15888561

[B15] van Janssen DK Heylen M Mets T Verbeelen D Evaluation of functional and mental state and quality of life in chronic haemodialysis patients Int Urol Nephrol 2004 36 263 267 15368708 10.1023/b:urol.0000034653.59183.77

[B16] Wong CF McCarthy M Howse ML Williams PS Factors affecting survival in advanced chronic kidney disease patients who choose not to receive dialysis Ren Fail 2007 29 653 659 10.1080/08860220701459634 17763158

[B17] Hamaker ME Buurman BM van Munster BC Smorenburg C de Rooij SE The value of a comprehensive geriatric assessment for patient care in acutely hospitalized older patients with cancer Oncologist 2011 16 1403 1412 10.1634/theoncologist.2010-0433 21914699 PMC3228071

[B18] Kruizenga HM De JP Seidell JC Neelemaat F van Bodegraven AA Wierdsma NJ Are malnourished patients complex patients? Health status and care complexity of malnourished patients detected by the Short Nutritional Assessment Questionnaire (SNAQ) Eur J Intern Med 2006 17 189 194 10.1016/j.ejim.2005.11.019 16618452

[B19] Collins SL Moore RA McQuay HJ The visual analogue pain intensity scale: what is moderate pain in millimetres? Pain 1997 72 95 97 10.1016/S0304-3959(97)00005-5 9272792

[B20] Schoonhoven L Grobbee DE Donders AR Algra A Grypdonck MH Bousema MT Prediction of pressure ulcer development in hospitalized patients: a tool for risk assessment Qual Saf Health Care 2006 15 65 70 10.1136/qshc.2005.015362 16456213 PMC2563999

[B21] Charlson ME Pompei P Ales KL MacKenzie CR A new method of classifying prognostic comorbidity in longitudinal studies: development and validation J Chronic Dis 1987 40 373 383 10.1016/0021-9681(87)90171-8 3558716

[B22] Katz S Ford AB Moskowitz RW Jackson BA Jaffe MW Studies of illness in the aged. the index of adl: a standardized measure of biological and psychosocial function JAMA 1963 185 914 919 10.1001/jama.1963.03060120024016 14044222

[B23] Weinberger M Samsa GP Schmader K Greenberg SM Carr DB Wildman DS Comparing proxy and patients' perceptions of patients' functional status: results from an outpatient geriatric clinic J Am Geriatr Soc 1992 40 585 588 1587975 10.1111/j.1532-5415.1992.tb02107.x

[B24] Krabbe PF Stouthard ME Essink-Bot ML Bonsel GJ The effect of adding a cognitive dimension to the EuroQol multiattribute health-status classification system J Clin Epidemiol 1999 52 293 301 10.1016/S0895-4356(98)00163-2 10235169

[B25] Hoeymans N van LH Westert GP The health status of the Dutch population as assessed by the EQ-6D Qual Life Res 2005 14 655 663 10.1007/s11136-004-1214-z 16022059

[B26] Folstein MF Folstein SE McHugh PR "Mini-mental state". A practical method for grading the cognitive state of patients for the clinician J Psychiatr Res 1975 12 189 198 10.1016/0022-3956(75)90026-6 1202204

[B27] Jorm AF Jacomb PA The Informant Questionnaire on Cognitive Decline in the Elderly (IQCODE): socio-demographic correlates, reliability, validity and some norms Psychol Med 1989 19 1015 1022 10.1017/S0033291700005742 2594878

[B28] de Jonghe JF Schmand B Ooms ME Ribbe MW Abbreviated form of the Informant Questionnaire on cognitive decline in the elderly Tijdschr Gerontol Geriatr 1997 28 224 229 9526793

[B29] Cummings JL Mega M Gray K Rosenberg-Thompson S Carusi DA Gornbein J The Neuropsychiatric Inventory: comprehensive assessment of psychopathology in dementia Neurology 1994 44 2308 2314 10.1212/WNL.44.12.2308 7991117

[B30] Sheikh J Yesavage J Geriatric Depression Scale (GDS): Recent evidence and development of a shorter version Clinical Gerontologist: The Journalof Aging and Mental Health 1986 5 165 173

[B31] Inouye SK van Dyck CH Alessi CA Balkin S Siegal AP Horwitz RI Clarifying confusion: the confusion assessment method. A new method for detection of delirium Ann Intern Med 1990 113 941 948 2240918 10.7326/0003-4819-113-12-941

[B32] Pot AM Van DR Deeg DJ [Perceived stress caused by informal caregiving. Construction of a scale] Tijdschr Gerontol Geriatr 1995 26 214 219 8750982

[B33] De Jong Gierveld J Van Tilburg T A shortened scale for overall, social and emotional loneliness Tijdschr Gerontol Geriatr 2008 39 4 15 10.1007/BF03078118 18365511

[B34] Genestier S Meyer N Chantrel F Alenabi F Brignon P Maaz M Prognostic survival factors in elderly renal failure patients treated with peritoneal dialysis: a nine-year retrospective study Perit Dial Int 2010 30 218 226 10.3747/pdi.2009.00043 20124194

[B35] Joly D Anglicheau D Alberti C Octogenarians reaching end-stage renal disease: cohort study of decision-making and clinical outcomes J Am Soc Nephrol 2003 14 1012 1021 10.1097/01.ASN.0000054493.04151.80 12660336

[B36] Munikrishnappa D Chronic kidney disease (CKD) in the elderly – a geriatrician's perspective Aging Male 2007 10 113 137 10.1080/13685530701419096 17701657

[B37] Li M Porter E Lam R Jassal SV Quality improvement through the introduction of interdisciplinary geriatric hemodialysis rehabilitation care Am J Kidney Dis 2007 50 90 97 10.1053/j.ajkd.2007.04.011 17591528 PMC7115717

[B38] Jassal SV Watson D Dialysis in late life: benefit or burden Clin J Am Soc Nephrol 2009 4 2008 2012 10.2215/CJN.04610709 19965545

[B39] Lopes AA Bragg J Young E Goodkin D Mapes D Combe C Depression as a predictor of mortality and hospitalization among hemodialysis patients in the United States and Europe Kidney Int 2002 62 199 207 10.1046/j.1523-1755.2002.00411.x 12081579

[B40] Thong MS Kaptein AA Krediet RT Boeschoten EW Dekker FW Social support predicts survival in dialysis patients Nephrol Dial Transplant 2007 22 845 850 10.1093/ndt/gfl700 17164318

[B41] Untas A Thumma J Rascle N Rayner H Mapes D Lopes AA The associations of social support and other psychosocial factors with mortality and quality of life in the dialysis outcomes and practice patterns study Clin J Am Soc Nephrol 2011 6 142 152 10.2215/CJN.02340310 20966121 PMC3022236

[B42] Arnold RM Zeidel ML Dialysis in frail elders–a role for palliative care N Engl J Med 2009 361 1597 1598 10.1056/NEJMe0907698 19828538

[B43] Maas HA Janssen-Heijnen ML Olde Rikkert MG Machteld Wymenga AN Comprehensive geriatric assessment and its clinical impact in oncology Eur J Cancer 2007 43 2161 2169 10.1016/j.ejca.2007.08.002 17855074

[B44] Terret C Zulian GB Naiem A Albrand G Multidisciplinary approach to the geriatric oncology patient J Clin Oncol 2007 25 1876 1881 10.1200/JCO.2006.10.3291 17488986

[B45] Wedding U Rohrig B Klippstein A Pientka L Hoffken K Age, severe comorbidity and functional impairment independently contribute to poor survival in cancer patients J Cancer Res Clin Oncol 2007 133 945 950 10.1007/s00432-007-0233-x 17534661 PMC12160824

[B46] Rao AV Hsieh F Feussner JR Cohen HJ Geriatric evaluation and management units in the care of the frail elderly cancer patient J Gerontol A Biol Sci Med Sci 2005 60 798 803 10.1093/gerona/60.6.798 15983186

[B47] Murtagh FE Addington-Hall JM Higginson IJ End-stage renal disease: a new trajectory of functional decline in the last year of life J Am Geriatr Soc 2011 59 304 308 10.1111/j.1532-5415.2010.03248.x 21275929

